# Frontotemporal Lobar Degeneration and MicroRNAs

**DOI:** 10.3389/fnagi.2016.00017

**Published:** 2016-02-09

**Authors:** Paola Piscopo, Diego Albani, Anna E. Castellano, Gianluigi Forloni, Annamaria Confaloni

**Affiliations:** ^1^Department of Neuroscience, Istituto Superiore di SanitàRome, Italy; ^2^Department of Neuroscience, Istituto di Ricerche Farmacologiche Mario NegriMilano, Italy; ^3^Department of Neurology, Neuromed InstitutePozzilli, Italy

**Keywords:** miRNA, frontotemporal lobar degeneration, progranulin, TDP43, social behavioral deficits

## Abstract

Frontotemporal lobar degeneration (FTLD) includes a spectrum of disorders characterized by changes of personality and social behavior and, often, a gradual and progressive language dysfunction. In the last years, several efforts have been fulfilled in identifying both genetic mutations and pathological proteins associated with FTLD. The molecular bases undergoing the onset and progression of the disease remain still unknown. Recent literature prompts an involvement of RNA metabolism in FTLD, particularly microRNAs (miRNAs). Dysregulation of miRNAs in several disorders, including neurodegenerative diseases, and increasing importance of circulating miRNAs in different pathologies has suggested to implement the study of their possible application as biological markers and new therapeutic targets; moreover, miRNA-based therapy is becoming a powerful tool to deepen the function of a gene, the mechanism of a disease, and validate therapeutic targets. Regarding FTLD, different studies showed that miRNAs are playing an important role. For example, several reports have evaluated miRNA regulation of the progranulin gene suggesting that it is under their control, as described for miR-29b, miR-107, and miR-659. More recently, it has been demonstrated that *TMEM106B* gene, which protein is elevated in FTLD-TDP brains, is repressed by miR-132/212 cluster; this post-transcriptional mechanism increases intracellular levels of progranulin, affecting its pathways. These findings if confirmed could suggest that these microRNAs have a role as potential targets for some related-FTLD genes. In this review, we focus on the emerging roles of the miRNAs in the pathogenesis of FTLD.

## Introduction

Frontotemporal lobar degeneration (FTLD) is a pathological process that represents one of the main causes of dementia (Cardarelli et al., 2010), after *Alzheimer's* Disease (AD), accounting for 5–10% of all dementias and is characterized by atrophy in the frontal and temporal lobes of the brain (Seltman and Matthews, 2012). At clinical level, FTLD patients manifest personality and social behavior changes and, often gradual and progressive language dysfunction (McKhann et al., 2001). Approximately 15% of patients also develop symptoms of motor neuron dysfunction. Pathologically, around 40% of FTLD patients present with inclusions of hyperphosphorylated microtubule-associated protein tau (FTLD-tau; Morris et al., 2001), whereas the most FTLD patients shows ubiquitin-positive inclusions (FTLD-U) constituted mainly by TAR DNA-binding protein 43 (TDP-43; Neumann et al., 2006). Genetically, several genes are described in association to the disease, including microtubule associated protein tau (*MAPT*), progranulin (*GRN*), and *C9ORF72*. Rare mutations were also found in genes coding charged multivesicular body protein 2B (*CHMP2B*), the valosin-containing protein (*VCP*), TAR DNA-binding protein (*TARDBP*), and *FUS* (Sieben et al., 2012). The genetic heterogeneity is further complicated by possible different clinical manifestations within a single mutation and even within individual families, suggesting the involvement of post-transcriptional regulation mechanisms such as microRNAs (miRNAs).

## miRNA biogenesis and functions

Aberrant RNA processing can cause or exacerbate neurodegenerative diseases *via* many mechanisms. Less than 5% of total cellular RNA is messenger RNA coding for proteins, and the other 95% is non-coding RNAs that has been shown to have a profound impact on gene expression regulation and also on other neurochemical processes, and to be implicated as complexity multipliers in both normal and abnormal conditions of the human central nervous system (Nelson and Keller, 2007; Peterson et al., 2009; Rapoport and Nelson, 2011). miRNAs are members of non-coding RNAs and silence mRNA molecules via base-pairing with their complementary sequences (Fire et al., 1998). miRNAs are involved in all development and pathologic processes targeting protein-coding transcripts mainly in the 3′UTR (Lytle et al., 2007). The biogenesis of miRNAs is controlled at temporal and spatial level. miRNAs are composed of ~22 nucleotides and produced by two RNase III proteins: Drosha and Dicer. Indeed, after the transcription, primary miRNAs (pri-miRNA) undergo to following steps: Drosha cleaves at the base of a stem to generate ~60–100 nt hairpin precursor miRNAs (pre-miRNA; Lee et al., 2003; Denli et al., 2004); then, pre-miRNA is carried into the cytoplasm by exportin 5 (Exp5) and once in the cytoplasm, it is processed by Dicer, arising a mature duplex of miRNAs long about 22 bp (Cullen, 2004; Winter and Diederichs, 2011).

### FTLD pathogenically-related proteins and miRNA biogenesis

TDP-43, one of FTLD pathogenically-related proteins, binds both DNA and RNA and has multiple functions in mRNA metabolism contributing to transcriptional repression, pre-mRNA splicing and translational regulation (Buratti et al., 2001; Wang et al., 2008a; Da Cruz and Cleveland, 2011). Interestingly, TDP-43 has been found in a macromolecular complex containing Drosha and Dicer (Gregory et al., 2004; Kawahara and Mieda-Sato, 2012). In addition, TDP-43 binds directly to a subset of pri-miRNAs to promoting the production of pre-miRNAs (Kawahara and Mieda-Sato, 2012). FUS, another FTLD pathogenically-related protein, is a highly conserved RNA/DNA binding protein, as well. It participates to regulation of gene expression and RNA processing to the DNA damage response (Wang et al., 2013). FUS was also found in the macromolecular complex containing Drosha (Kawahara and Mieda-Sato, 2012) and binding to pri-miRNAs for miRNA processing (Morlando et al., 2012).

## miRNAs in the nervous system

miRNAs are abundant in the nervous system and show a brain-specific expression profile correlated to the expression of their targets. In fact, as suggested by several microarray studies, the brain expresses a large number of miRNAs that participate in nervous system physiology (Lagos-Quintana et al., 2002; Miska et al., 2004; Lim et al., 2005; Manakov et al., 2009). In particular, they have key roles in the regulation of different biological functions, as important mediators of plasticity and neurogenesis, in which they channelize cellular physiology toward neuronal differentiation. Moreover, they regulate the proliferation and self-renewal of neural stem cells indirectly influencing neurogenesis (Stappert et al., 2015). miRNAs are dysregulated in several neurodegenerative diseases that share a final common pathway of neuronal cell death. Neurodegeneration includes complex pathogenic mechanisms; above all, aging is the main risk factor, but other common characteristics exist among these pathologies, such as neuroinflammation, protein aggregation, and mitochondrial dysfunction. Profiling analysis of miRNA expression in several nervous system disorders identified signatures correlated with the diagnosis, progression and prognosis of diseases, and treatment monitoring (Shafi et al., 2010; Langbaum et al., 2013). The role of specific miRNAs in a disease has been established in just a few cases and most of the mechanistic data originates from invertebrate model systems. In any case, the importance of miRNAs in neurodegenerative disorders and their emerging role in AD, Parkinson's disease, Huntington's disease, and Amyotrophic lateral sclerosis is becoming more and more evident. (Kim et al., 2007; Johnson et al., 2008; Wang et al., 2008b; Nunez-Iglesias et al., 2010; Lukiw et al., 2012; Grasso et al., 2015). In AD patients, for example, profiling studies highlighted dramatic changes in several miRNAs (i.e., miR-29 cluster, miR-107, miR-9, miR-125b, and miR-128; Delay et al., 2012). Although these alterations under pathological conditions should be interpreted with caution, they support the idea that dysregulation of miRNAs is a common mechanism in neurodegenerative diseases. Consequently, the study of miRNAs is a novel tool to understanding these diseases and possibly find therapeutic targets. For a complete coverage of the roles of miRNAs in other neurodegenerative diseases, we suggest some recent review articles (Gascon and Gao, 2012; Maciotta et al., 2013; Grasso et al., 2014; Femminella et al., 2015).

## miRNAs and FTLD

In the last years, several studies have been addressed to understand the role of miRNAs in the pathophysiology of FTLD. Two approaches are frequently used to study the involvement of miRNAs in the pathology. The first is based on a global approach of miRNA profiling with subsequent validation by RT-qPCR. The second approach is based on the analysis of miRNAs showing a direct connection with the disease.

### miRNAs expression profiling

Kocerha and colleagues decribed an expression profiling study to identify miRNAs responsive to *GRN* haploinsufficiency. They analyzed the global miRNAs in the frontal cortex of 8 FTLD-TDP patients with *GRN* mutations and compared them to a population of 32 FTLD-TDP patients with no apparent genetic abnormalities (Kocerha et al., 2011). Using TaqMan Array Human MicroRNA, they identified 20 miRNAs differentially expressed (*P* < 0.05) in frontal cortex. Moreover, a validation study by RT-qPCR analyzes confirmed a differential expression for 9 of the 20 miRNAs in frontal cortex (miR-33a^*^, let7i^*^, miR-516a-3p, miR-548b-5p, miR-548c-5p, miR-565, miR-571, miR-572, and miR-922). Since these data have not been functionally validated, the mechanism by which PGRN haploinsufficiency in FTLD patients leads to altered expression of these miRNAs is currently unclear and requires future studies. Moreover, little or nothing has been studied about the role of these miRNAs in brain or other neurodegenerative diseases. For example, miR-922 is described as a promoter of tau phosphorylation by down-regulating ubiquitin carboxy-terminal hydrolase L1 expression in AD (Zhao et al., 2014), but other studies are needed to explain their role on neurological disease pathogenesis.

An expression profiling study was also performed by RNA deep sequencing in miRNAs derived from well-characterized brain samples originated from autopsy series at the University of Kentucky AD Center (UK ADC; Hébert et al., 2013). They analyzed temporal neocortex gray matter samples of five FTLD cases, as well as non-demented controls (*n* = 2), AD (*n* = 5), dementia with Lewy bodies (*n* = 4), hippocampal sclerosis in aging (*n* = 4). From a total of 795 miRNAs expressed in the human brain, they identified 31 human miRNAs differently expressed. The validation study by miRNA RT-qPCR analysis reported a down-regulation of miR-132-3p in FTLD cases. A similar result was obtained by the same authors in AD patients, as well suggesting that this miRNA could have a more general function in neurodegeneration than a direct role in specific subtypes of pathology. Recently, this miRNA was found participating to a post-transcriptional mechanism that increases intracellular levels of PGRN, as reported below.

### miRNA-132, miR-29b, and miR-659 regulate GRN expression

Progranulin (PGRN), encoded by the *GRN* gene, is a widely expressed protein involved in the regulation of cell growth and cell cycle progression (Bateman et al., 1990; He and Bateman, 2003). Mutations in the *GRN* gene are linked with FTLD (Baker et al., 2006). Recent findings suggest that *GRN* is under the control of miRNAs (Table 1).

**Table 1:** **miRNAs involved in FTLD**.

**Alterated miRNAs**	**Regulation**	**Target genes**	**Related pathway to FTD**	**References**
miR-132/212 Cluster	Downregulated in FTLD-TDP brains	TMEM106B	Regulating PGRN levels	Chen-Plotkin et al., 2012; Hébert et al., 2013
miR-107	Downregulated in mice with cortical traumatic brain injury	GRN	Regulating PGRN levels	Wang et al., 2010
miR-659	Genetic variant located in the 3′-UTR of GRN within a miR-659 binding site	GRN	Genetic variability in binding-site of miR-659 can increase the risk for FTLD	Rademakers et al., 2008
miR-29b	–	GRN	Regulating PGRN levels	Jiao et al., 2010
miR-9 and pri-miR-9-2	Downregulated in patient neurons	Neuronal specification genes	Neuronal toxicity	Zhang et al., 2013
	Downregulated in TDP-43 mutants of Drosophila			Li et al., 2013
miR-124	Downregulated in a FTD mouse model	AMPAR	Regulating social behavior	Gascon et al., 2014

#### miR-132 and miR-132-3p cluster

miR-132 down-regulation was also recently observed in another cohort of FTLD-TDP brains (Baker et al., 2006), thus confirming the data from Hebert and colleagues (He and Bateman, 2003). This miRNA is a member of the miR-132 cluster (miR-132, miR-132^*^, and miR-212). miR-132 and miR-212 target *TMEM106B* through two specific sites in the *TMEM106B* 3′UTR. Chen Plotkin and colleagues, having observed increased *TMEM106B* expression in FTLD-TDP, supposed a possible upstream role for miR-132/212. Effectively, they showed first of all that miR-132 and miR-212 normally repress *TMEM106B* and then that they are decreased in FTLD-TDP. Moreover, an involvement of *TMEM106B* gene on *GRN* expression was also observed; in details, this gene was found to be linked to FTLD-TDP (Van Deerlin et al., 2010; Van der Zee et al., 2011) and *TMEM106B* risk genotypes correlated with decreased plasma progranulin levels (Finch et al., 2011). Overall, these results suggest a model in which decreased levels of miR-132/212 carry out to a higher *TMEM106B* expression leading to increased *TMEM106B* expression and to endosomal-lysosomal dysfunction, which may, in turn, further increase the levels of TMEM106B, and perturb PGRN pathways, thereby increasing the risk of developing FTLD-TDP (Chen-Plotkin et al., 2012).

Further evidence linking miR-132 to FTLD are the reports addressing its role to the regulate splicing process of the microtubule associated protein tau gene (Smith et al., 2011; Hebert et al., 2012) and an involvement in cognitive function in experimental models including neuronal/synaptic integrity and the brain's response to stressors (O'Neill, 2009; Mellios et al., 2011; Miller et al., 2012; Wanet et al., 2012; Shaltiel et al., 2013; Bicker et al., 2014).

#### miR-107

A miRNA assay (RIP-Chip) in neuron-like human cell lines showed that *GRN* is a strong target for miR-107 (Wang et al., 2010). This miRNA recognizes mainly sequence elements in the open reading frame rather than the 3′UTR of *GRN* mRNA; these sequences are highly conserved among vertebrate species. Wang and colleagues described a down-regulation of miR-107 in a mouse model of traumatic brain injury, speculating that this miRNA plays a role in modulating neuronal repair and regeneration in the mammalian brain through molecular regulation of *GRN* and other mRNA targets. Moreover, they described that miR-107 has also been involved in AD. In addition, Noren Hooten et al. compared miRNA expression of blood mononuclear cells in young individuals related to old individuals by RT-qPCR analysis to test possible aging-related differences and found that miR-107 was significantly decreased in aged individuals (Noren Hooten et al., 2010). Taken to in consideration this evidence, it would be interesting to study the existence of a possible link between miR-107 expression and FTLD.

#### miR-659

Another miRNA regulating *GRN* expression is miR-659. Rademakers and colleagues described an allelic variant associated to FTLD (Rademakers et al., 2008) located in the 3′UTR of *GRN* corresponding to a predicted binding site for miR-659. They described that genotype TT for SNP rs5848 was associated to FTLD with a 3.2-fold increased risk to develop the pathology suggesting that a genetic variant in a miRNA binding-site can influence the risk for FTLD. They further demonstrate that miR-659 can regulate GRN expression *in vitro*, with miR-659 binding more efficiently to the high risk T allele of rs5848. In order to evaluate the possibility that rs5848 variant is involved in *GRN* regulation in patients, they analyzed its expression in FTLD patient brains revealing an association between *GRN* lower levels and the TT genotype. However, other studies are needed to verify a possible role of miR-659 in the brain and particularly in FTLD.

#### miR-29b

Another binding site in the 3′UTR of *GRN* mRNA was identified for miR-29b. Indeed, Jiao and colleagues demonstrated that miR-29b interacts directly with the *GRN* 3′UTR, regulating the *GRN* expression (Jiao et al., 2010). MiR-29b has a role in neurodevelopment and reach the highest level in adult mouse brain. Moreover, it is differentially expressed in AD patients (Hebert et al., 2008), even if to date we do not have evidence about a regulatory role of this miRNA in FTLD patients. Thus, several miRNAs may contribute to the pathogenesis of FTLD linked to progranulin deficiency.

### miR-9 and TDP43

As previously reported, TDP-43 has two RNA recognition motifs and is involved in several roles of RNA metabolism (Da Cruz and Cleveland, 2011; Lee et al., 2011). Among these, TDP43 has been associated to miRNA processing; miR-9 is one of these and is described as an evolutively conserved miRNA, brain-specific and implicated in some neurodegenerative diseases. A work published by Zhang and colleagues described a down-regulation of miR-9 in iPSC-derived neurons (iPSC: induced pluripotent stem cells) of FTD/ALS patients with *TDP-43* mutations; they explained their results as an a effect of decreased TDP-43 expression revealed in these neurons (Zhang et al., 2013). Moreover, they found that levels of both pre-miR-9-2 and pri-miR-9-2 were also reduced, suggesting that miRNA decrease did not seem to be due to a defect in the miRNA-processing pathway.

Recently, a work on mutant *TDP-43* showed an involvement in FTLD of Drosha protein, one of the most important miRNA processing molecules involved in the biogenesis of miRNAs. In this study Drosha increased in correlation with a TDP-43 activation in Neuro 2A cells, leading to hypothesize that a mutant *TDP-43* in FTLD-U, associated with Drosha instability, can induce neuronal toxicity (Kim et al., 2015). Also in Drosophila, TDP-43 seems to regulate miR-9a levels by interacting with pri-miR-9a and likely promoting its stability (Li et al., 2013).

### miR-124 and social behavioral deficits

miR-124 is one of most abundant miRNAs in the brain known to be involved in neuronal development. (Gao, 2010). Furthermore, it seems to have a role also in neurodegeneration and in regulating social behavior in FTLD models. Gascon and colleagues studied a new mouse model of FTLD exhibiting deficits in sociability (Gascon et al., 2014). They found that miR-124 is down-regulated in this model causing a dysregulation in AMPA receptor (AMPAR) composition and a selective impairment in sociability. Moreover, they examined miR-124 and AMPAR expression in subjects with FTLD, focusing on the frontal cortex in the subset of cases with bvFTD, whose clinical presentation is closest to the phenotypes observed in their mouse model. They found a decrease in miR-124 expression and a concomitant up-regulation of two AMPAR subunits in the frontal cortex of bvFTD patients compared with age-matched controls. Furthermore, they studied miR-124 and AMPARs in established iPSC lines derived from subjects with bvFTD (Almeida et al., 2012, 2013), in which the expression of miR-124 and AMPARs in 8-week-old neurons were reduced and some AMPAR subunit mRNAs upregulated.

Increasing evidences support the presence of common miRNAs differentially expressed in several neurodegenerative diseases. For example, miR-132, decreased in FTLD brains, is emerging as gradually down-regulated during AD at early, mid, and late stages of disease and showing its important role in the maintenance of brain integrity (Lau et al., 2014). Another miRNA regulated in both FTLD and AD is miR-107 that decreases in the temporal cortex of LOAD patients; in luciferase assays, it showed a functional interaction with 3′UTR region of BACE1, a protease involved in the cleavage of APP for the generation of amyloid-beta (Wang et al., 2008b). miR-107 also controls the expression of other proteins relevant to AD pathology, such as cofilin (Yao et al., 2010), an actin-binding protein that accumulates in cytoplasmic inclusions. Also miR-29b was found to be alterated in AD and interacting with BACE, as described by Hebert and colleagues (Hebert et al., 2008). Moreover, it was hypothesized that deregulation of miR-29b in the brain was associated to the increase of apoptotic markers in AD, since miR-29b has been shown to target a family of pro-apoptotic regulators (Delay et al., 2012). Then, it could be conceivable the existence of common miRNAs pathways involved in the molecular mechanisms of some neurodegenerative diseases.

## Final remarks

In the last years, miRNAs studies are becoming more and more powerful to understand molecular mechanisms of complex disorders, such as dementias because they can regulate in fine-manner the pathways involved in the onset and progression of the disease and represent molecular tools as biomarkers and new therapeutic targets. Recent literature prompts that miRNAs could have an upstream and/or downstream role in the frontotemporal pathology to regulate a cascade of events leading to the neurodegeneration.

The studies mentioned in this mini-review provide interesting clues about miRNAs involvement in the pathology, and even if rather fragmentary, they could be further deepened by using high-throughput techniques such as small-RNAseq possibly not only in patients, but also in cellular and animal models. Several advances have been performed on the developing of FTLD models, such as tau and TDP-43 transgenic mice reproducing some features of FTLD, such as reduced survival, fragmentation and insolubility of protein aggregates, gliosis, and neuronal loss. Moreover, iPSCs are likely to become a standard in the field of neurodegenerative diseases, complementing, but not replacing, genetic animal models (Ittner et al., 2015). Methodologies used for miRNAs in FTLD ranged from Northern blotting to microarrays, but further advances could arrive from small RNASeq sequencing useful to detect small RNAs from very low amounts of starting biological material and discovery novel miRNAs expressed in a specific pathology.

Despite last advances on molecular and pathological bases in FTLD, the pathology remains still orphan for disease-modifying therapies. The identification of disease modulating factors will in turn ameliorate the knowledge of FTLD molecular pathways opening, in the future, to new therapeutic strategies.

## Author contributions

PP, DA: Substantial contribution to the conception or organization of the manuscript; AEC and GF: Drafting the work and revising it critically; and AC: Final approval of the version to be published.

### Conflict of interest statement

The authors declare that the research was conducted in the absence of any commercial or financial relationships that could be construed as a potential conflict of interest.

## References

[B1] Almeida S. Gascon E. Tran H. Chou H. J. Gendron T. F. Degroot S.. (2013). Modeling key pathological features of frontotemporal dementia with C9ORF72 repeat expansion in iPSC-derived human neurons. Acta Neuropathol. 126, 385–399. 10.1007/s00401-013-1149-y 23836290 PMC3753484

[B2] Almeida S. Zhang Z. Coppola G. Mao W. Futai K. Karydas A.. (2012). Induced pluripotent stem cell models of progranulin-deficient frontotemporal dementia uncover specific reversible neuronal defects. Cell Rep. 2, 789–798. 10.1016/j.celrep.2012.09.007 23063362 PMC3532907

[B3] Baker M. Mackenzie I. R. Pickering-Brown S. M. Gass J. Rademakers R. Lindholm C.. (2006). Mutations in progranulin cause tau-negative frontotemporal dementia linked to chromosome 17. Nature 442, 916–919. 10.1038/nature05016 16862116

[B4] Bateman A. Belcourt D. Bennett H. Lazure C. Solomon S. (1990). Granulins, a novel class of peptide from leukocytes. Biochem. Biophys. Res. Commun. 173, 1161–1168. 10.1016/S0006-291X(05)80908-8 2268320

[B5] Bicker S. Lackinger M. Weiß K. Schratt G. (2014). MicroRNA-132, -134, and -138: a microRNA troika rules in neuronal dendrites. Cell. Mol. Life Sci. 71, 3987–4005. 10.1007/s00018-014-1671-7 25008044 PMC11113804

[B6] Buratti E. Dork T. Zuccato E. Pagani F. Romano M. Baralle F. E. (2001). Nuclear factor TDP-43 and SR proteins promote *in vitro* and *in vivo* CFTR exon 9 skipping. EMBO J. 20, 1774–1784. 10.1093/emboj/20.7.1774 11285240 PMC145463

[B7] Cardarelli R. Kertesz A. Knebl J. A. (2010). Frontotemporal dementia: a review for primary care physicians. Am. Fam. Physician 82, 1372–1377. 21121521

[B8] Chen-Plotkin A. S. Unger T. L. Gallagher M. D. Bill E. Kwong L. K. Volpicelli-Daley L.. (2012). TMEM106B, the risk gene for frontotemporal dementia, is regulated by the microRNA-132/212 cluster and affects progranulin pathways. J. Neurosci. 32, 11213–11227. 10.1523/JNEUROSCI.0521-12.2012 22895706 PMC3446826

[B9] Cullen B. R. (2004). Derivation and function of small interfering RNAs and microRNAs. Virus Res. 102, 3–9. 10.1016/j.virusres.2004.01.009 15068874

[B10] Da Cruz S. Cleveland D. W. (2011). Understanding the role of TDP-43 and FUS/TLS in ALS and beyond. Curr. Opin. Neurobiol. 21, 904–919. 10.1016/j.conb.2011.05.029 21813273 PMC3228892

[B11] Delay C. Mandemakers W. Hébert S. S. (2012). MicroRNAs in *Alzheimer's* disease. Neurobiol. Dis. 46, 285–290. 10.1016/j.nbd.2012.01.003 22285895

[B12] Denli A. M. Tops B. B. Plasterk R. H. Ketting R. F. Hannon G. J. (2004). Processing of primary microRNAs by the microprocessor complex. Nature 432, 231–235. 10.1038/nature03049 15531879

[B13] Femminella G. D. Ferrara N. Rengo G. (2015). The emerging role of microRNAs in *Alzheimer's* disease. Front. Physiol. 6:40. 10.3389/fphys.2015.00040 25729367 PMC4325581

[B14] Finch N. Carrasquillo M. M. Baker M. Rutherford N. J. Coppola G. Dejesus-Hernandez M.. (2011). TMEM106B regulates progranulin levels and the penetrance of FTLD in GRN mutation carriers. Neurology 76, 467–474. 10.1212/WNL.0b013e31820a0e3b 21178100 PMC3034409

[B15] Fire A. Xu S. Montgomery M. K. Kostas S. A. Driver S. E. Mello C. C. (1998). Potent and specific genetic interference by double-stranded RNA in Caenorhabditis elegans. Nature 391, 806–811. 9486653 10.1038/35888

[B16] Gao F. B. (2010). Context-dependent functions of specific microRNAs in neuronal development. Neural Dev. 5:25. 10.1186/1749-8104-5-25 20920300 PMC2958854

[B17] Gascon E. Gao F. B. (2012). Cause or effect: misregulation of microRNA pathways in neurodegeneration. Front. Neurosci. 6:48. 10.3389/fnins.2012.00048 22509148 PMC3321503

[B18] Gascon E. Lynch K. Ruan H. Almeida S. Verheyden J. M. Seeley W. W.. (2014). Alterations in microRNA-124 and AMPA receptors contribute to social behavioral deficits in frontotemporal dementia. Nat. Med. 20, 1444–1451. 10.1038/nm.3717 25401692 PMC4257887

[B19] Grasso M. Piscopo P. Confaloni A. Denti M. A. (2014). Circulating miRNAs as biomarkers for neurodegenerative disorders. Molecules 19, 6891–6910. 10.3390/molecules19056891 24858274 PMC6271879

[B20] Grasso M. Piscopo P. Crestini A. Confaloni A. Denti M. A. (2015). Circulating microRNAs in neurodegenerative diseases. EXS 106, 151–169. 10.1007/978-3-0348-0955-9_7 26608203

[B21] Gregory R. I. Yan K. P. Amuthan G. Chendrimada T. Doratotaj B. Cooch N.. (2004). The microprocessor complex mediates the genesis of microRNAs. Nature 432, 235–240. 10.1038/nature03120 15531877

[B22] Hebert S. S. Horrè K. Nicolaï L. Papadopoulou A. S. Mandemakers W. Silahtaroglu A. N.. (2008). Loss of microRNA cluster miR-29a/b-1 in sporadic *Alzheimer's* disease correlates with increased BACE1/beta-secretase expression. Proc. Natl. Acad. Sci. U.S.A. 105, 6415–1520. 10.1073/pnas.0710263105 18434550 PMC2359789

[B23] Hebert S. S. Sergeant N. Buee L. (2012). MicroRNAs and the regulation of tau metabolism. Int. J. Alzheimers Dis. 2012:406561. 10.1155/2012/406561 22720189 PMC3374946

[B24] Hébert S. S. Wang W. X. Zhu Q. Nelson P. T. (2013). A study of small RNAs from cerebral neocortex of pathology-verified *Alzheimer*'s disease, dementia with lewy bodies, hippocampal sclerosis, frontotemporal lobar dementia, and non-demented human controls. J. Alzheimers Dis. 35, 335–348. 10.3233/JAD-122350 23403535 PMC3753694

[B25] He Z. Bateman A. (2003). Progranulin (granulin-epithelin precursor, PC-cell-derived growth factor, acrogranin) mediates tissue repair and tumorigenesis. J. Mol. Med. 81, 600–612. 10.1007/s00109-003-0474-3 12928786

[B26] Ittner L. M. Halliday G. M. Kril J. J. Götz J. Hodges J. R. Kiernan M. C. (2015). FTD and ALS–translating mouse studies into clinical trials. Nat. Rev. Neurol. 11, 360–366. 10.1038/nrneurol.2015.65 25939274

[B27] Jiao J. Herl L. D. Farese R. V. Gao F. B. (2010). MicroRNA-29b regulates the expression level of human progranulin, a secreted glycoprotein implicated in frontotemporal dementia. PLoS ONE 5:e10551. 10.1371/journal.pone.0010551 20479936 PMC2866661

[B28] Johnson R. Zuccato C. Belyaev N. D. Guest D. J. Cattaneo E. Buckley N. J. (2008). A microRNA-based gene dysregulation pathway in Huntington's disease. Neurobiol. Dis. 29, 438–445. 10.1016/j.nbd.2007.11.001 18082412

[B29] Kawahara Y. Mieda-Sato A. (2012). TDP-43 promotes microRNA biogenesis as a component of the Drosha and Dicer complexes. Proc. Natl. Acad. Sci. U.S.A. 109, 3347–3352. 10.1073/pnas.1112427109 22323604 PMC3295278

[B30] Kim J. Inoue K. Ishii J. Vanti W. B. Voronov S. V. Murchison E.. (2007). A MicroRNA feedback circuit in midbrain dopamine neurons. Science 317, 1220–1224. 10.1126/science.1140481 17761882 PMC2782470

[B31] Kim K. Y. Lee H. W. Shim Y. M. Mook-Jung I. Jeon G. S. Sung J. J. (2015). A phosphomimetic mutant TDP-43 (S409/410E) induces Drosha instability and cytotoxicity in Neuro 2A cells. Biochem. Biophys. Res. Commun. 464, 236–243. 10.1016/j.bbrc.2015.06.125 26102026

[B32] Kocerha J. Kouri N. Baker M. Finch N. DeJesus-Hernandez M. Gonzalez J.. (2011). Altered microRNA expression in frontotemporal lobar degeneration with TDP-43 pathology caused by progranulin mutations. BMC Genomics 12:527. 10.1186/1471-2164-12-527 22032330 PMC3229715

[B33] Lagos-Quintana M. Rauhut R. Yalcin A. Meyer J. Lendeckel W. Tuschl T. (2002). Identification of tissue-specific microRNAs from mouse. Curr. Biol. 12, 735–739. 10.1016/S0960-9822(02)00809-6 12007417

[B34] Langbaum J. B. Fleisher A. S. Chen K. Ayutyanont N. Lopera F. Quiroz Y. T.. (2013). Ushering in the study and treatment of preclinical *Alzheimer* disease. Nat. Rev. Neurol. 9, 371–381. 10.1038/nrneurol.2013.107 23752908 PMC4084675

[B35] Lau P. Frigerio C. S. De Strooper B. (2014). Variance in the identification of microRNAs deregulated in *Alzheimer's* disease and possible role of lincRNAs in the pathology: the need of larger datasets. Ageing Res. Rev. 17, 43–53. 10.1016/j.arr.2014.02.006 24607832

[B36] Lee E. B. Lee V. M. Trojanowski J. Q. (2011). Gains or losses: molecular mechanisms of TDP43-mediated neurodegeneration. Nat. Rev. Neurosci. 13, 38–50. 10.1038/nrn3121 22127299 PMC3285250

[B37] Lee Y. Ahn C. Han J. Choi H. Kim J. Yim J.. (2003). The nuclear RNase III Drosha initiates microRNA processing. Nature 425, 415–419. 10.1038/nature01957 14508493

[B38] Li Z. Lu Y. Xu X. L. Gao F. B. (2013). The FTD/ALS-associated RNA-binding protein TDP-43 regulates the robustness of neuronal specification through microRNA-9a in *Drosophila*. Hum. Mol. Genet. 22, 218–225. 10.1093/hmg/dds420 23042786 PMC3526156

[B39] Lim L. P. Lau N. C. Garrett-Engele P. Grimson A. Schelter J. M. Castle J.. (2005). Microarray analysis shows that some microRNAs downregulate large numbers of target mRNAs. Nature 433, 769–773. 10.1038/nature03315 15685193

[B40] Lukiw W. J. Alexandrov P. N. Zhao Y. (2012). Spreading of *Alzheimer's* disease inflammatory signaling through soluble micro-RNA. Neuroreport 23, 621–626. 10.1097/WNR.0b013e32835542b0 22660168 PMC4467540

[B41] Lytle J. R. Yario T. A. Steitz J. A. (2007). Target mRNAs are repressed as efficiently by microRNA-binding sites in the 5′UTR as in the 3'UTR. Proc. Natl. Acad. Sci. U.S.A. 104, 9667–9672. 10.1073/pnas.0703820104 17535905 PMC1887587

[B42] Maciotta S. Meregalli M. Torrente Y. (2013). The involvement of microRNAs in neurodegenerative diseases. Front. Cell. Neurosci. 7:265. 10.3389/fncel.2013.00265 24391543 PMC3867638

[B43] Manakov S. A. Grant S. G. Enright A. J. (2009). Reciprocal regulation of microRNA and mRNA profiles in neuronal development and synapse formation. BMC Genomics 10:419. 10.1186/1471-2164-10-419 19737397 PMC2759968

[B44] McKhann G. M. Albert M. S. Grossman M. Miller B. Dickson D. Trojanowski J. Q. (2001). Work Group on frontotemporal dementia and pick's disease. clinical and pathological diagnosis of frontotemporal dementia: report of the Work Group on *Frontotemporal Dementia and Pick's Disease*. Arch. Neurol. 58, 1803–1809. 11708987 10.1001/archneur.58.11.1803

[B45] Mellios N. Sugihara H. Castro J. Banerjee A. Le C. Kumar A.. (2011). miR-132, an experience-dependent microRNA, is essential for visual cortex plasticity. Nat. Neurosci. 14, 1240–1242. 10.1038/nn.2909 21892155 PMC3183341

[B46] Miller B. H. Zeier Z. Xi L. Lanz T. A. Deng S. Strathmann J.. (2012). MicroRNA-132 dysregulation in schizophrenia has implications for both neurodevelopment and adult brain function. Proc. Natl. Acad. Sci. U.S.A. 109, 3125–3130. 10.1073/pnas.1113793109 22315408 PMC3286960

[B47] Miska E. A. Alvarez-Saavedra E. Townsend M. Yoshii A. Sestan N. Rakic P.. (2004). Microarray analysis of microRNA expression in the developing mammalian brain. Genome Biol. 5:R68 10.1186/gb-2004-5-9-r68 15345052 PMC522875

[B48] Morlando M. Dini Modigliani S. Torrelli G. Rosa A. Di Carlo V. Caffarelli E.. (2012). FUS stimulates microRNA biogenesis by facilitating co-transcriptional Drosha recruitment. EMBO J. 31, 4502–4510. 10.1038/emboj.2012.319 23232809 PMC3545295

[B49] Morris H. R. Khan M. N. Janssen J. C. Brown J. M. Perez-Tur J. Baker M.. (2001). The genetic and pathological classification of familial frontotemporal dementia. Arch. Neurol. 58, 1813–1816. 11708988 10.1001/archneur.58.11.1813

[B50] Nelson P. T. Keller J. N. (2007). RNA in brain disease: no longer just “the messenger in the middle.” J. Neuropathol. Exp. Neurol. 66, 461–468. 10.1097/01.jnen.0000240474.27791.f3 17549006

[B51] Neumann M. Sampathu D. M. Kwong L. K. Truax A. C. Micsenyi M. C. Chou T. T.. (2006). Ubiquitinated TDP-43 in frontotemporal lobar degeneration and amyotrophic lateral sclerosis. Science 314, 130–133. 10.1126/science.1134108 17023659

[B52] Noren Hooten N. Abdelmohsen K. Gorospe M. Ejiogu N. Zonderman A. B. Evans M. K. (2010). MicroRNA expression patterns reveal differential expression of target genes with age. PLoS ONE 5:e10724. 10.1371/journal.pone.0010724 20505758 PMC2873959

[B53] Nunez-Iglesias J. Liu C. C. Morgan T. E. Finch C. E. Zhou X. J. (2010). Joint genome-wide profiling of miRNA and mRNA expression in *Alzheimer's* disease cortex reveals altered miRNA regulation. PLoS ONE 5:e8898. 10.1371/journal.pone.0008898 20126538 PMC2813862

[B54] O'Neill L. A. (2009). Boosting the brain's ability to block inflammation via microRNA-132. Immunity 31, 854–855. 10.1016/j.immuni.2009.11.004 20064444

[B55] Peterson K. J. Dietrich M. R. McPeek M. A. (2009). MicroRNAs and metazoan macroevolution: insights into canalization, complexity, and the Cambrian explosion. Bioessays 31, 736–747. 10.1002/bies.200900033 19472371

[B56] Rademakers R. Eriksen J. L. Baker M. Robinson T. Ahmed Z. Lincoln S. J.. (2008). Common variation in the miR-659 binding-site of GRN is a major risk factor for TDP43-positive frontotemporal dementia. Hum. Mol. Genet. 17, 3631–3642. 10.1093/hmg/ddn257 18723524 PMC2581433

[B57] Rapoport S. I. Nelson P. T. (2011). Biomarkers and evolution in *Alzheimer* disease. Prog. Neurobiol. 95, 510–513. 10.1016/j.pneurobio.2011.07.006 21801803 PMC5878862

[B58] Seltman R. E. Matthews B. R. (2012). Frontotemporal lobar degeneration: epidemiology, pathology, diagnosis and management. CNS Drugs 26, 841–870. 10.2165/11640070-000000000-00000 22950490

[B59] Shafi G. Aliya N. Munshi A. (2010). Micro RNA signatures in neurological disorders. Can. J. Neurol. Sci. 37, 177–185. 10.1017/S0317167100009902 20437927

[B60] Shaltiel G. Hanan M. Wolf Y. Barbash S. Kovalev E. Shoham S. (2013). Hippocampal microRNA-132 mediates stress-inducible cognitive deficits through its acetyl-cholinesterase target. Brain Struct. Funct. 218, 59–72. 10.1007/s00429-011-0376-z 22246100 PMC3535403

[B61] Sieben A. Van Langenhove T. Engelborghs S. Martin J. J. Boon P. Cras P.. (2012). The genetics and neuropathology of frontotemporal lobar degeneration. Acta Neuropathol. 124, 353–372. 10.1007/s00401-012-1029-x 22890575 PMC3422616

[B62] Smith P. Y. Delay C. Girard J. Papon M. A. Planel E. Sergeant N.. (2011). MicroRNA-132 loss is associated with tau exon 10 inclusion in progressive supranuclear palsy. Hum. Mol. Genet. 20, 4016–4024. 10.1093/hmg/ddr330 21807765

[B63] Stappert L. Roese-Koerner B. Brüstle O. (2015). The role of microRNAs in human neural stem cells, neuronal differentiation and subtype specification. Cell Tissue Res. 359, 47–64. 10.1007/s00441-014-1981-y 25172833 PMC4284387

[B64] Van Deerlin V. M. Sleiman P. M. Martinez-Lage M. Chen-Plotkin A. Wang L. S. Graff-Radford N. R.. (2010). Common variants at 7p21 are associated with frontotemporal lobar degeneration with TDP-43 inclusions. Nat. Genet. 42, 234–239. 10.1038/ng.536 20154673 PMC2828525

[B65] Van der Zee J. Van Langenhove T. Kleinberger G. Sleegers K. Engelborghs S. Vandenberghe R.. (2011). TMEM106B is associated with frontotemporal lobar degeneration in a clinically diagnosed patient cohort. Brain 134, 808–815. 10.1093/brain/awr007 21354975 PMC3044834

[B66] Wanet A. Tacheny A. Arnould T. Renard P. (2012). miR-212/ 132 expression and functions: within and beyond the neuronal compartment. Nucleic Acids Res. 40, 4742–4753. 10.1093/nar/gks151 22362752 PMC3367188

[B67] Wang I. F. Wu L. S. Chang H. Y. Shen C. K. (2008a). TDP-43, the signature protein of FTLD-U, is a neuronal activity-responsive factor. J. Neurochem. 105, 797–806. 18088371 10.1111/j.1471-4159.2007.05190.x

[B68] Wang W. X. Rajeev B. W. Stromberg A. J. Ren N. Tang G. Huang Q.. (2008b). The expression of microRNA miR-107 decreases early in *Alzheimer's* disease and may accelerate disease progression through regulation of beta-site amyloid precursor protein-cleaving enzyme 1. J. Neurosci. 28, 1213–1223. 10.1523/JNEUROSCI.5065-07.2008 18234899 PMC2837363

[B69] Wang W. X. Wilfred B. R. Madathil S. K. Tang G. Hu Y. Dimayuga J.. (2010). miR-107 regulates granulin/progranulin with implications for traumatic brain injury and neurodegenerative disease. Am. J. Pathol. 177, 334–345. 10.2353/ajpath.2010.091202 20489155 PMC2893676

[B70] Wang W. Y. Pan L. Su S. C. Quinn E. J. Sasaki M. Jimenez J. C.. (2013). Interaction of FUS and HDAC1 regulates DNA damage response and repair in neurons. Nat Neurosci. 16, 1383–1391. 10.1038/nn.3514 24036913 PMC5564396

[B71] Winter J. Diederichs S. (2011). MicroRNA Northern blotting, precursor cloning, and Ago2-improved RNA interference. Methods Mol. Biol. 676, 85–100. 10.1007/978-1-60761-863-8_7 20931392

[B72] Yao J. Hennessey T. Flynt A. Lai E. Beal M. F. Lin M. T. (2010). MicroRNA-related cofilin abnormality in *Alzheimer's* disease. PLoS ONE 5:e15546. 10.1371/journal.pone.0015546 21179570 PMC3002958

[B73] Zhang Z. Almeida S. Lu Y. Nishimura A. L. Peng L. Sun D.. (2013). Downregulation of microRNA-9 in iPSC-derived neurons of FTD/ALS patients with TDP-43 mutations. PLoS ONE 8:e76055. 10.1371/journal.pone.0076055 24143176 PMC3797144

[B74] Zhao Z. B. Wu L. Xiong R. Wang L. L. Zhang B. Wang C.. (2014). MicroRNA-922 promotes tau phosphorylation by downregulating ubiquitin carboxy-terminal hydrolase L1 (UCHL1) expression in the pathogenesis of *Alzheimer's* disease. Neuroscience 275, 232–237. 10.1016/j.neuroscience.2014.06.013 24950120

